# Supplementary material for: Potential impact of annual vaccination with reformulated COVID-19 vaccines: lessons from the U.S. COVID-19 Scenario Modeling Hub
Source: medRxiv. 2023 Oct 26:2023.10.26.23297581. Preprint. [Version 1] doi: 10.1101/2023.10.26.23297581 (PMC10635209; doi:10.1101/2023.10.26.23297581)
Supplement: Supplementary file 1 [file NIHPP2023.10.26.23297581v1-supplement-1.pdf]

medRxiv preprint doi: https://doi.org/10.1101/2023.10.26.23297581; this version posted October 26, 2023. The copyright holder for this preprint (which was not certified by peer review) is the author/funder, who has granted medRxiv a license to display the preprint in perpetuity.
It is made available under a CC-BY 4.0 International license .

Supplementary figures

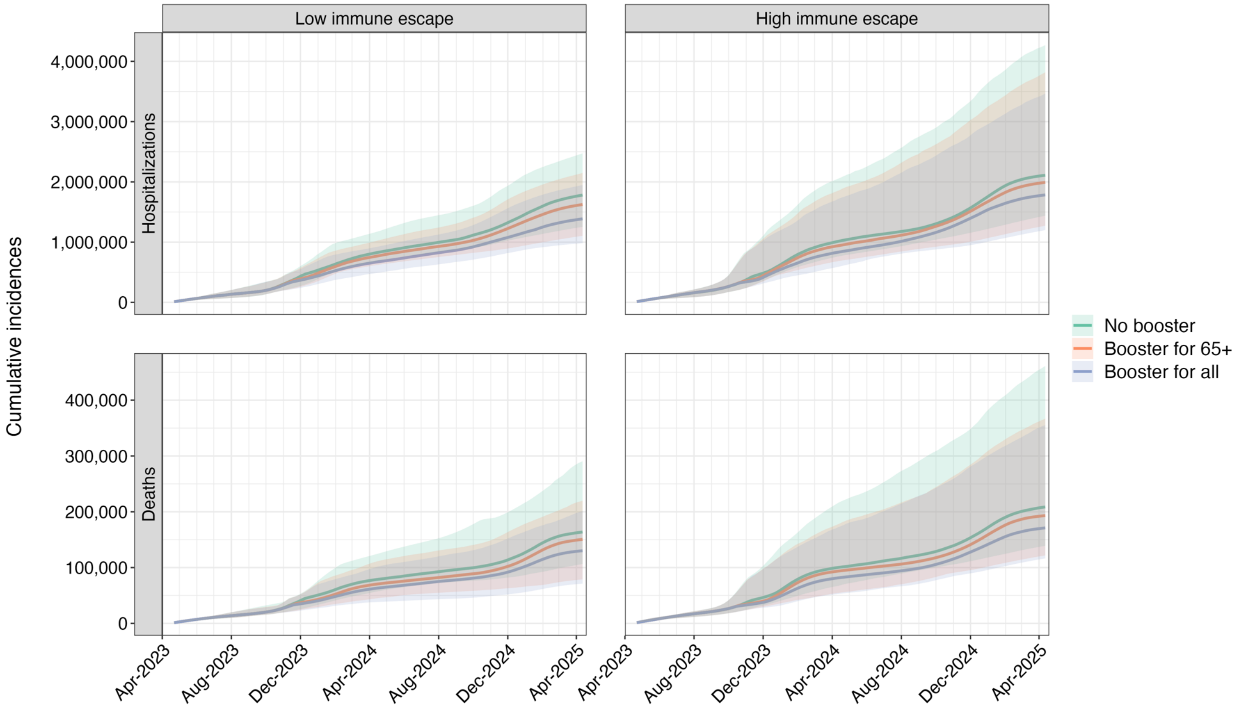

Figure S1: Projected cumulative COVID-19 hospitalizations and deaths in the United States by scenario, April 2023–April 2025.

Ensemble projections for cumulative COVID-19 hospitalization and deaths in the United States for the next two years (April 2023–April 2025) are shown by scenario. Lines and shades indicate the median of projected outcomes and 90% projection intervals. Each color represents different annual vaccination recommendations (no recommendation, reformulated vaccines recommended for those aged 65 and above, and recommended for all age groups).

medRxiv preprint doi: https://doi.org/10.1101/2023.10.26.23297581; this version posted October 26, 2023. The copyright holder for this preprint (which was not certified by peer review) is the author/funder, who has granted medRxiv a license to display the preprint in perpetuity.
It is made available under a CC-BY 4.0 International license .

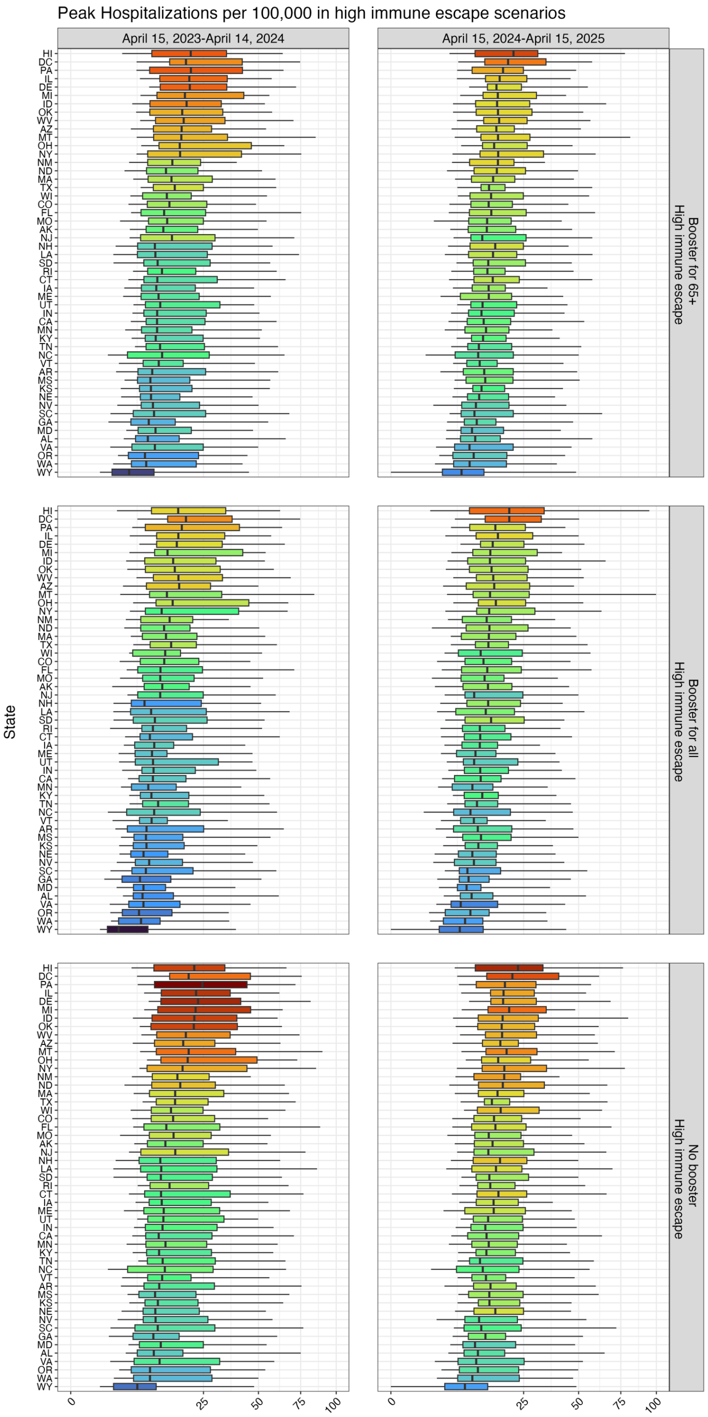

Figure S2: State-level peak COVID-19 hospitalizations in high immune escape scenarios by season and vaccination scenario.

The peak hospitalizations per 100,000 over the next two years (April 2023–April 2025) under high immune escape assumption are shown by US state and by vaccination scenario (no recommendation, reformulated vaccines recommended for those aged 65 and above, and recommended for all age groups). Shades of blue indicate states with lower values and shades of red indicate states with higher values. For visualizations, square root scaling was applied in x-axes.

medRxiv preprint doi: https://doi.org/10.1101/2023.10.26.23297581; this version posted October 26, 2023. The copyright holder for this preprint (which was not certified by peer review) is the author/funder, who has granted medRxiv a license to display the preprint in perpetuity.
It is made available under a CC-BY 4.0 International license .

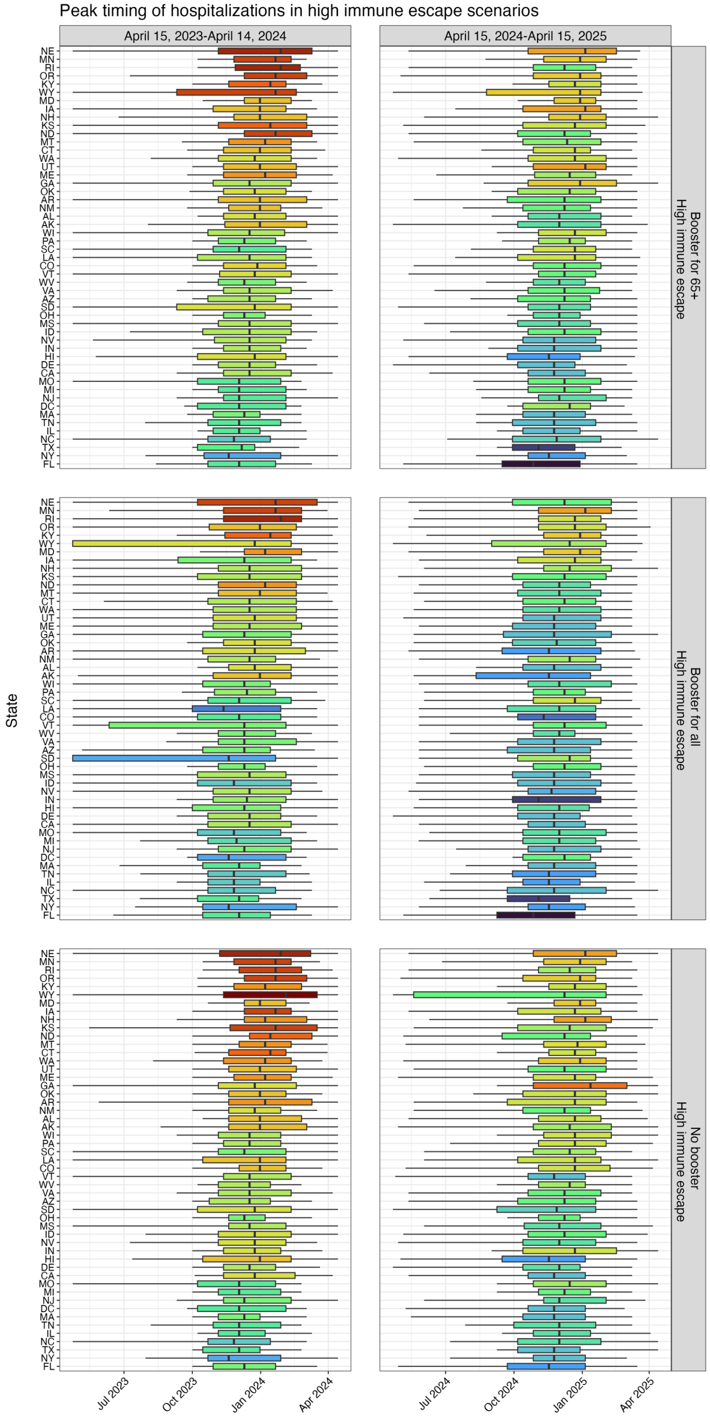

Figure S3: State-level peak timing of COVID-19 hospitalizations in high immune escape scenarios by season and vaccination scenario.

The peak timing of hospitalizations under high immune escape assumption is shown by US state and by vaccination scenario (no recommendation, reformulated vaccines recommended for those aged 65 and above, and recommended for all age groups). Shades of blue indicate states with lower values and shades of red indicate states with higher values.

medRxiv preprint doi: https://doi.org/10.1101/2023.10.26.23297581; this version posted October 26, 2023. The copyright holder for this preprint (which was not certified by peer review) is the author/funder, who has granted medRxiv a license to display the preprint in perpetuity.
It is made available under a CC-BY 4.0 International license .

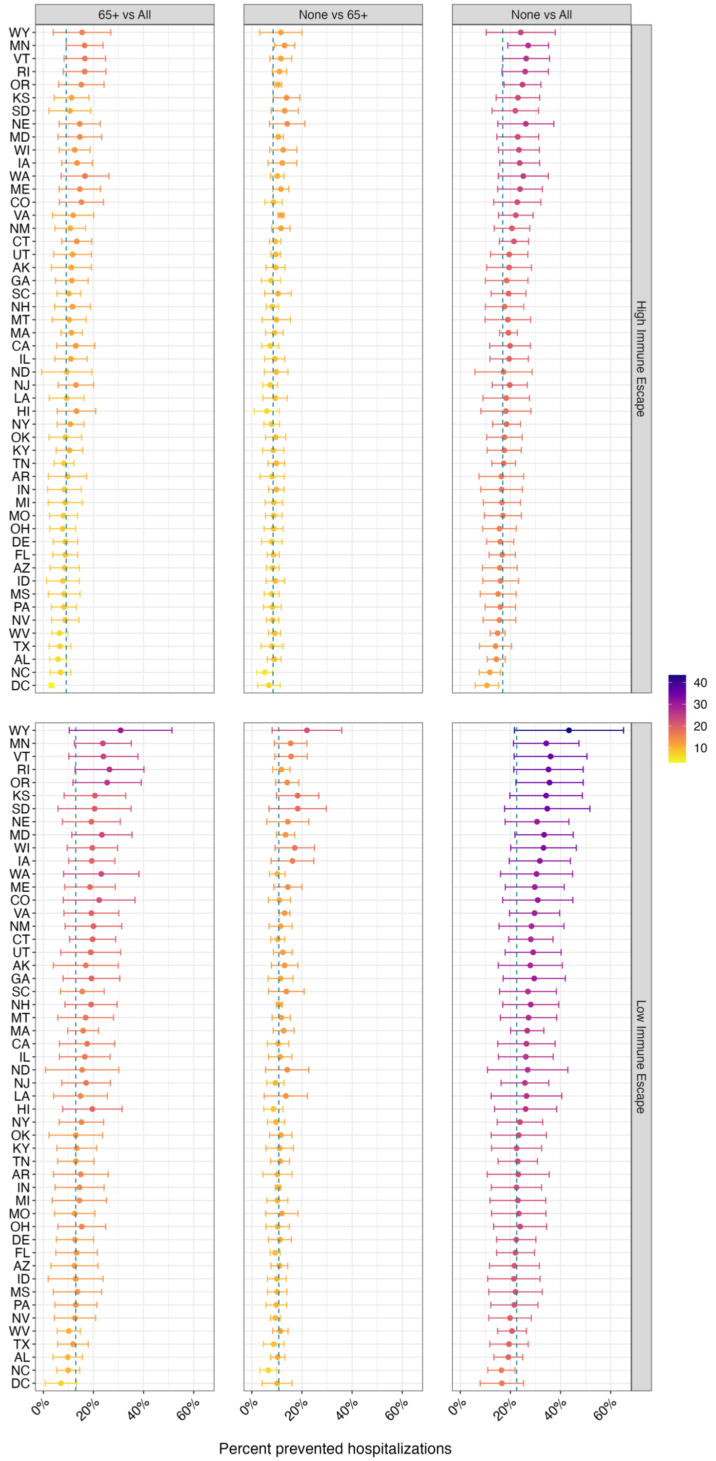

Figure S4: State-level percent prevented COVID-19 hospitalizations between the annual vaccination scenarios from April 2023 to April 2025 by scenario.

Relative differences in cumulative COVID-19 hospitalizations over the next two years (April 2023–April 2025) between different vaccination scenarios are shown by immune escape level and by US state. Shades of yellow indicate states with lower values and shades of purple indicate states with higher values.

medRxiv preprint doi: https://doi.org/10.1101/2023.10.26.23297581; this version posted October 26, 2023. The copyright holder for this preprint
(which was not certified by peer review) is the author/funder, who has granted medRxiv a license to display the preprint in perpetuity.
It is made available under a CC-BY 4.0 International license .

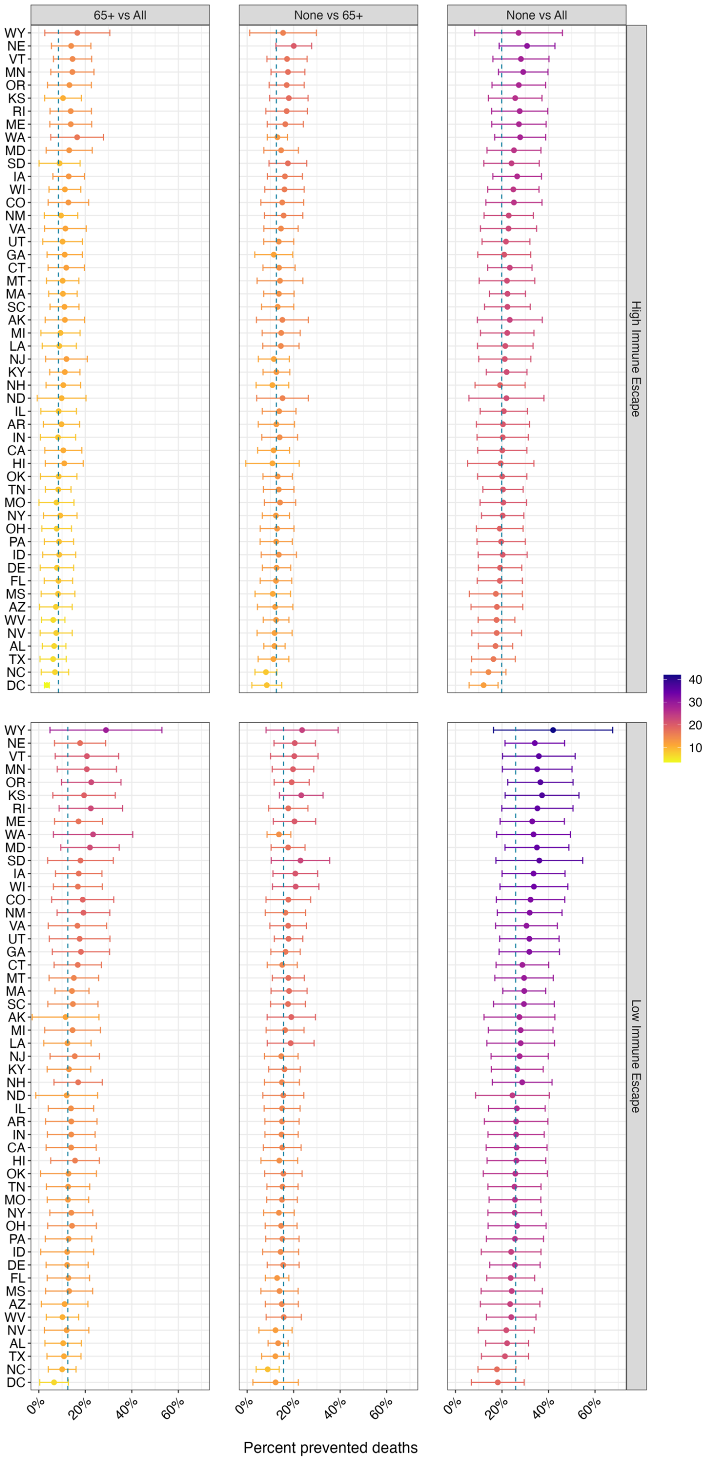

Figure S5: State-level percent prevented COVID-19 deaths between the annual vaccination scenarios from April 2023 to April 2025 by scenario.
Relative differences in cumulative COVID-19 deaths over the next two years (April 2023–April 2025) between different vaccination scenarios are shown by immune escape level and by US state. Shades of yellow indicate states with lower values and shades of purple indicate states with higher values.
